# Persistent hepatocyte apoptosis promotes tumorigenesis from diethylnitrosamine-transformed hepatocytes through increased oxidative stress, independent of compensatory liver regeneration

**DOI:** 10.1038/s41598-021-83082-7

**Published:** 2021-02-09

**Authors:** Yasutoshi Nozaki, Hayato Hikita, Satoshi Tanaka, Kenji Fukumoto, Makiko Urabe, Katsuhiko Sato, Yuta Myojin, Akira Doi, Kazuhiro Murai, Sadatsugu Sakane, Yoshinobu Saito, Takahiro Kodama, Ryotaro Sakamori, Tomohide Tatsumi, Tetsuo Takehara

**Affiliations:** Department of Gastroenterology and Hepatology, Osaka University Graduate School of Medicine, 2-2 Yamadaoka, Suita, Osaka Japan

**Keywords:** Hepatology, Liver diseases

## Abstract

Hepatocellular carcinoma highly occurs in chronic hepatitis livers, where hepatocyte apoptosis is frequently detected. Apoptosis is a mechanism that eliminates mutated cells. Hepatocyte apoptosis induces compensatory liver regeneration, which is believed to contribute to tumor formation. Hepatocyte-specific Mcl-1 knockout mice (Mcl-1^Δhep^ mice) developed persistent hepatocyte apoptosis and compensatory liver regeneration with increased oxidative stress in adulthood but had not yet developed hepatocyte apoptosis at the age of 2 weeks. When diethylnitrosamine (DEN) was administered to 2-week-old Mcl-1^Δhep^ mice, multiple liver tumors were formed at 4 months, while wild-type mice did not develop any tumors. These tumors contained the B-Raf V637E mutation, indicating that DEN-initiated tumorigenesis was promoted by persistent hepatocyte apoptosis. When N-acetyl-L-cysteine was given from 6 weeks of age, DEN-administered Mcl-1^Δhep^ mice had reduced oxidative stress and suppressed tumorigenesis in the liver but showed no changes in hepatocyte apoptosis or proliferation. In conclusion, enhanced tumor formation from DEN-transformed hepatocytes by persistent hepatocyte apoptosis is mediated by increased oxidative stress, independent of compensatory liver regeneration. For patients with livers harboring transformed cells, the control of oxidative stress may suppress hepatocarcinogenesis based on chronic liver injury.

## Introduction

Hepatocellular carcinoma (HCC) is the major form of primary liver cancer and is the sixth most commonly diagnosed cancer and the fourth leading cause of cancer-related deaths owing to its poor 5-year survival rate^1^. Approximately 30–40% of HCC patients are suitable to undergo potential curative therapies (e.g., surgical resection, radiofrequency ablation (RFA), transarterial chemoembolization (TACE) and liver transplantation); however, the remaining 60–70% of patients are eligible for only palliative treatment^2^. Although some molecular targeted drugs have been developed^3,4^, there is still a strong need for novel treatments or preventive strategies for HCC.

The strongest risk factor for HCC is chronic liver disease, a condition that is related to liver inflammation caused by exposure to one or several risk factors, including hepatitis B virus (HBV), hepatitis C virus (HCV), excessive consumption of alcohol, and metabolic syndrome^5^. Other cofactors, such as tobacco smoke inhalation and intake of aflatoxin B1, are well-characterized contributors to HCC^6^. Independent of the underlying etiology, all chronic liver diseases exhibit persistent hepatocyte damage with serum alanine aminotransferase (ALT) elevation. Hepatocyte apoptosis is a type of cell death frequently observed in human chronic liver diseases^7^. In livers with chronic liver diseases, various factors, such as oxidative stress, ER stress, HCV core protein, HBx protein or genome integration by HBV, induce the malignant transformation of hepatocytes^8–11^. These transformed cells should be eliminated by apoptosis to result in the suppression of tumor formation. On the other hand, hepatocyte apoptosis leads to compensatory liver regeneration, which is believed to contribute to tumor formation^12^. It is unclear whether hepatocyte apoptosis eventually suppresses or enhances tumor formation in livers harboring transformed cells.

Apoptosis was originally thought to have a tumor suppressor action to remove tumor cells, but since the enhancement of apoptosis in the liver causes compensatory liver regeneration, it was also related to the growth progression of tumor cells. Mcl-1 is an anti-apoptotic bcl-2 family proteins. In hepatocytes, Mcl-1 protects against mitochondrial pathway-induced apoptosis by inhibiting Bak/Bax activation through cooperation with Bcl-xL, another anti-apoptotic bcl-2 family proteins^13^. Hepatocyte-specific deficiency of Mcl-1 as well as Bcl-xL in mice results in persistent hepatocyte apoptosis by Bak/Bax activation in adulthood^13–15^. In the present study, we used hepatocyte-specific Mcl-1 knockout mice to evaluate the effect of hepatocyte apoptosis on tumor formation. Here, we show that the enhancement of oxidative stress associated with apoptosis may promote tumor progression independent of compensatory liver regeneration.

## Results

### DEN-induced tumor formation is promoted in Mcl-1^Δhep^ mice

**Figure 1:**
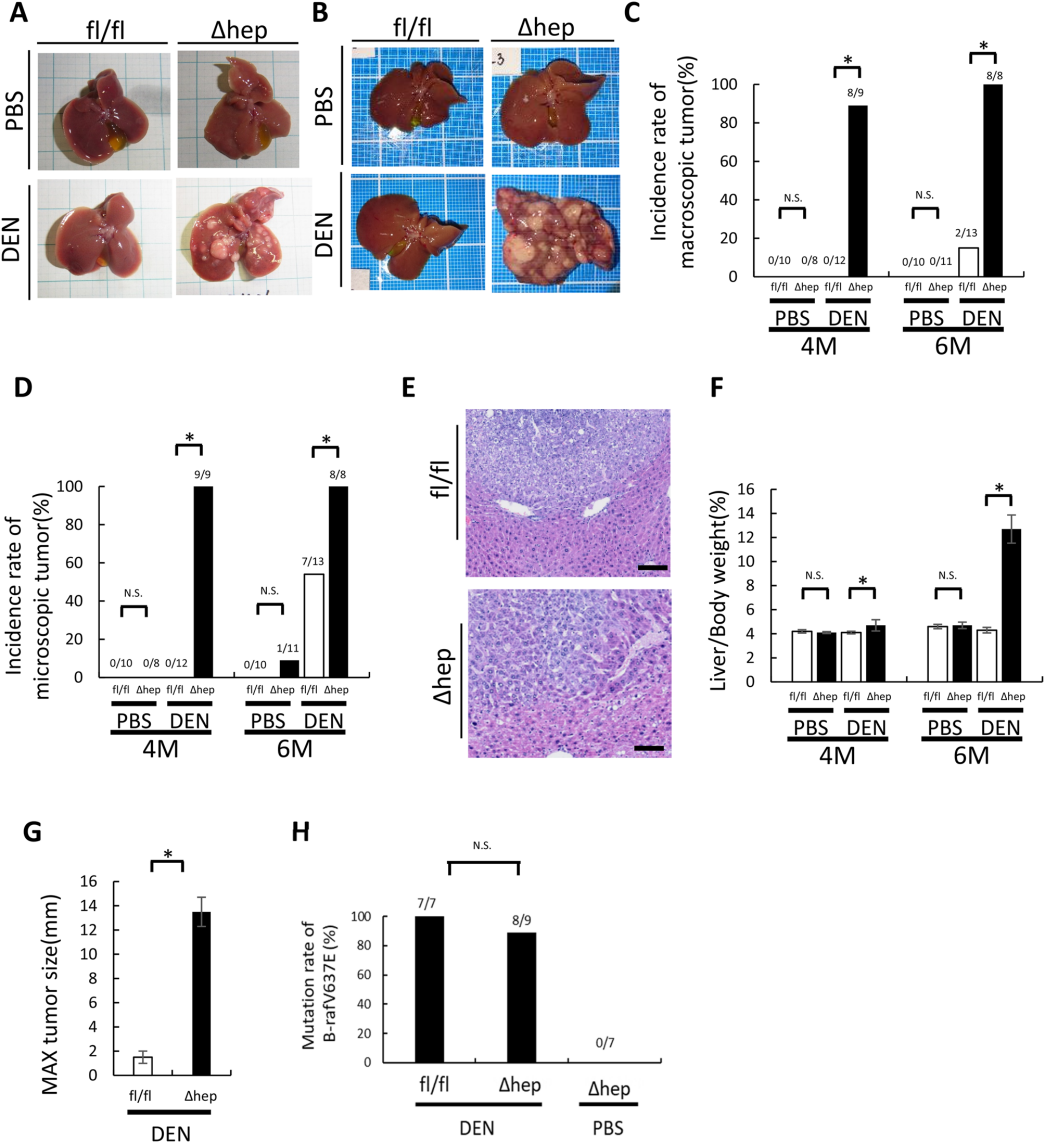
Mcl-1 deficiency enhanced DEN-induced liver tumor development. At the age of 2 weeks, male Mcl-1^fl/fl^ and Mcl-1^Δhep^ mice were intraperitonially injected with PBS or DEN (20 mg/kg BW), and their livers were harvested after 4 or 6 months. (**A**,**B**) Representative images of the livers of PBS- or DEN-treated male Mcl-1^ fl/fl^ and Mcl-1^Δhep^ mice at the ages of 4 months (**A**) and 6 months (**B**). (**C**,**D**) Incidence rate of macroscopic (**C**) or microscopic (**D**) tumors at the age of 4 and 6 months (n ≥ 8 per group, *chi-squared* test). (**E**) Representative H&E staining images of liver sections from the DEN-treated male 6-month-old Mcl-1^fl/fl^ and 6-month-old Mcl-1^Δhep^ mice. The scale bar is 100 µm. (**F**) Ratio of liver weight to body weight (mean ± SEM, *Mann–Whitney* test). (**G**) Maximum tumor size (diameters by caliper) at the age of 6 months (mean ± SEM, *Mann–Whitney* test). (**H**) Mutation rate of B-Raf V637E in liver tumors (*chi-squared* test, *p < 0.05).

To clarify the effect of hepatocyte apoptosis on tumor formation, we injected diethylnitrosamine (DEN) into two-week-old hepatocyte-specific Mcl-1 knockout mice (Mcl-1^Δhep^ mice), which showed persistent hepatocyte apoptosis in adulthood^13^. Six months after DEN injection, 54% (7/13) of the DEN-injected wild-type mice developed microscopic liver tumors, and 15% (2/13) of them developed macroscopic liver tumors (Fig. 1B,C,D,E). All developed liver tumors had the B-Raf V637E mutation (Fig. 1H), which is characteristic of DEN-induced liver tumors^16^. In contrast, all (8/8) DEN-injected Mcl-1^Δhep^ mice developed microscopic and macroscopic liver tumors (Fig. 1B,C,D,E). Most liver tumors also had the B-Raf V637E mutation (Fig. 1H). The liver/body weight ratio and maximum tumor size were significantly higher in the DEN-injected Mcl-1^Δhep^ mice than in the DEN-injected wild-type mice (Fig. 1F,G). Four months after DEN injection, the incidence rates of microscopic and macroscopic liver tumors were also higher in the DEN-injected Mcl-1^Δhep^ mice than in the DEN-injected wild-type mice (Fig. 1A,C,D). We previously reported that Mcl-1^Δhep^ mice developed liver tumors at the age of 1 year^17,18^. Indeed, one of eleven phosphate buffered saline (PBS)-injected Mcl-1^Δhep^ mice developed microscopic HCC 6 months after PBS injection (Fig. 1D). However, none of the liver tumors that developed in Mcl-1^Δhep^ mice at the age of 1 year had the B-Raf V637E mutation (Fig. 1H).

### Hepatocyte apoptosis and compensatory liver regeneration were observed in DEN-induced Mcl-1^Δhep^ mice after 4 weeks of age

**Figure 2:**
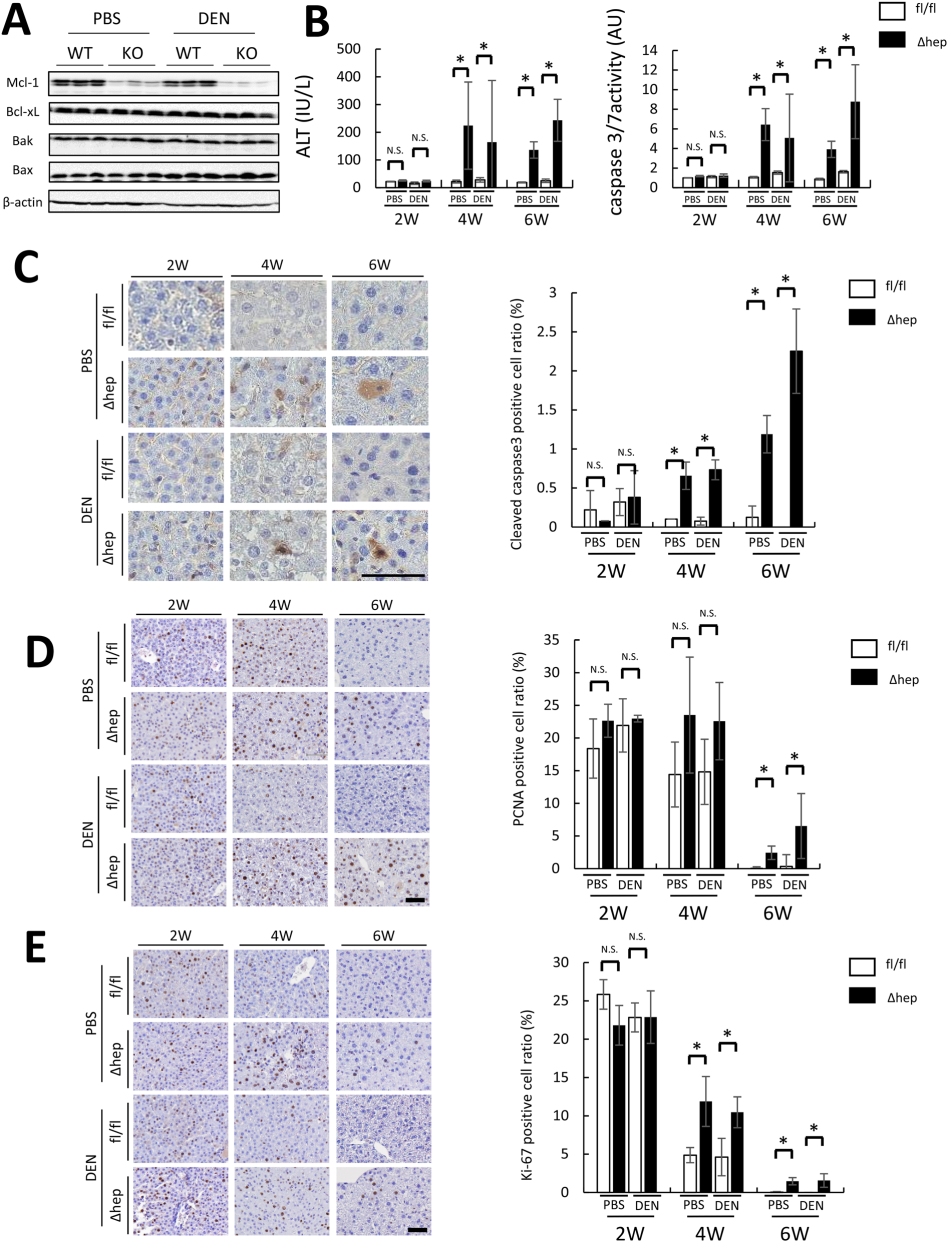
Mcl-1 deficiency resulted in increased hepatocyte apoptosis and subsequent compensatory liver regeneration after 4 weeks of age. Two-week-old male Mcl-1^fl/fl^ and Mcl-1^Δhep^ mice injected with PBS or DEN (20 mg/kg BW) were sacrificed at 2, 4 and 6 weeks of age. (**A**) Representative immunoblots of the indicated proteins in the liver lysates of two-week-old mice. Full-length blots/gels are presented in Supplementary Fig. 1. (**B**) Serum ALT levels and caspase 3/7 activity (n ≥ 8 per group, mean ± SEM, *Mann–Whitney* test). (**C**–**E**) Representative images of liver sections stained with anti-cleaved caspase-3 antibody (**C**), PCNA antibody, and Ki-67 antibody (**E**) (left) (n ≥ 4 per group). The scale bar is 50 µm. Each positive hepatocyte ratio was quantified and is shown as a percentage (right) (mean ± SEM, *Mann–Whitney* test, *p < 0.05).

Mcl-1 is an anti-apoptotic protein, and we previously reported that hepatocyte-specific Mcl-1 deficiency induced continuous hepatocyte apoptosis after 6 weeks of age, leading to the promotion of liver regeneration^13,17^. To elucidate the underlying mechanism of promoted tumor formation in DEN-treated Mcl-1^Δhep^ mice, we first examined hepatocyte apoptosis, liver regeneration and DNA damage after 2 weeks of age when DEN was administered. We confirmed that the expression levels of Mcl-1 decreased in Mcl-1^Δhep^ mice at the age of 2 weeks and that DEN treatment did not affect Mcl-1 expression (Fig. 2A). At the age of 2 weeks, there were no differences in the serum ALT levels, serum caspase-3/7 activity, or cleaved caspase-3-positive hepatocyte ratios between wild-type and Mcl-1^Δhep^ mice regardless of DEN administration (Fig. 2B,C), indicating that hepatocyte apoptosis was not enhanced in Mcl-1^Δhep^ mice. At the ages of 4 and 6 weeks, the cleaved caspase-3-positive hepatocyte ratios, serum ALT levels and caspase-3/7 activity were higher in Mcl-1^Δhep^ mice than in wild-type mice with or without DEN injection, indicating that Mcl-1 deficiency in hepatocytes induced persistent hepatocyte apoptosis after 4 weeks (Fig. 2B,C). There were no differences in the PCNA-positive hepatocyte ratios or Ki-67-positive hepatocyte rates between wild-type and Mcl-1^Δhep^ mice with or without DEN injection at the age of 2 weeks, while they were higher at the age of 6 weeks (Fig. 2D,E).

### Mcl-1^Δhep^ mice showed persistent DEN-induced DNA damage with increased hepatocyte oxidative stress

**Figure 3:**
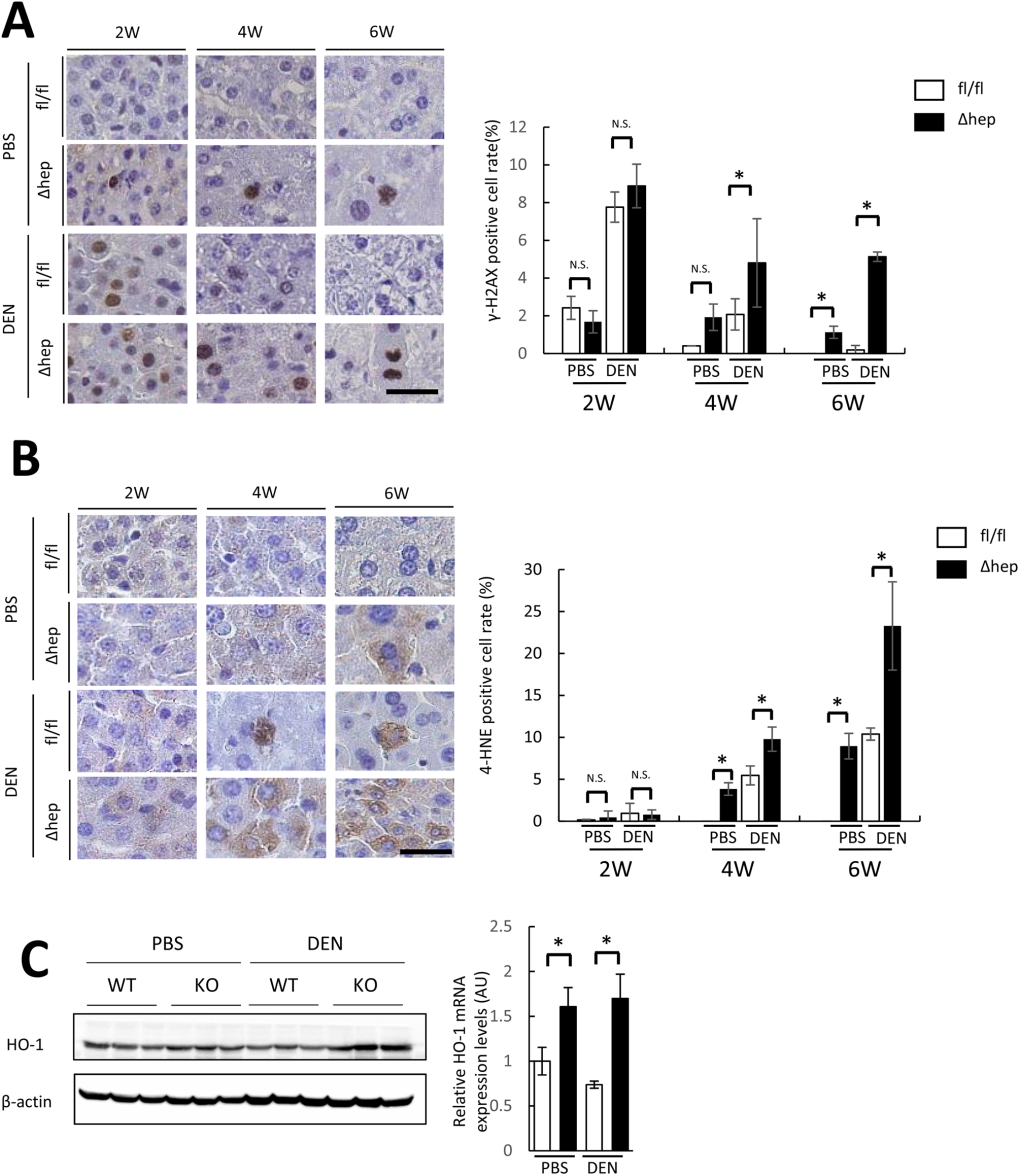
Mcl-1 deficiency impaired DNA damage repair with elevation of oxidative stress. Two-week-old male Mcl-1^fl/fl^ and Mcl-1^Δhep^ mice injected with PBS or DEN (20 mg/kg BW) were sacrificed at 2, 4 and 6 weeks of age. (**A**,**B**) Representative images of liver sections stained with anti-γ-H2AX (**A**) or anti-4-HNE (**B**) antibody (left). The scale bar is 50 µm. γ-H2AX-positive and 4-HNE-positive hepatocyte ratios were quantified and are shown as percentages (right) (n ≥ 4 per group, mean ± SEM, *Mann–Whitney* test, *p < 0.05). (**C**) Representative immunoblots of HO-1 in the liver of six-week-old mice (left), and expression levels of HO-1 by real- time PCR in the liver of six-week-old mice (right) (n = 8 per group, mean ± SEM, *Mann–Whitney* test, *p < 0.05). Full-length blots/gels are presented in Supplementary Fig. 2.

DEN is a potent alkylating agent that induces DNA damage upon conversion into alkyldiazohydroxide by cytochrome P450. DEN injection (2 weeks after birth) increased the positive hepatocyte ratio of γ-H2AX, which is one of central component in DNA repair and exerts its actions at nascent DNA double strand break sites^19^, regardless of the Mcl-1 genotype (Fig. 3A). DNA double strand break are repaired by DNA repair system^19^. The DEN-induced high γ-H2AX positive cell rate decreased in the time course (Fig. 3A), consistent with a previous report^20^. At 6 weeks of age, the positive hepatocyte ratio of γ-H2AX in the Mcl-1^Δhep^ mice was significantly higher than that in the wild-type mice (Fig. 3A).

Next, we evaluated oxidative stress in the liver, which was previously reported to increase in Mcl-1^Δhep^ mouse livers in adulthood^17^. The 4-hydroxy-2-nonenal (4-HNE)-positive hepatocyte ratio, a marker of lipid peroxidation, was significantly higher in the Mcl-1^Δhep^ mice than in the wild-type mice (Fig. 3B). The expression levels of heme oxygenase-1 (HO-1), which is induced by oxidative stress though Nrf2 activation^21^, were significantly higher in DEN-treated the Mcl-1^Δhep^ mice than in the DEN-treated wild-type mice (Fig. 3C).

### NAC treatment suppresses tumor formation in DEN-treated Mcl-1^Δhep^ mice with a reduction in DNA damage

**Figure 4:**
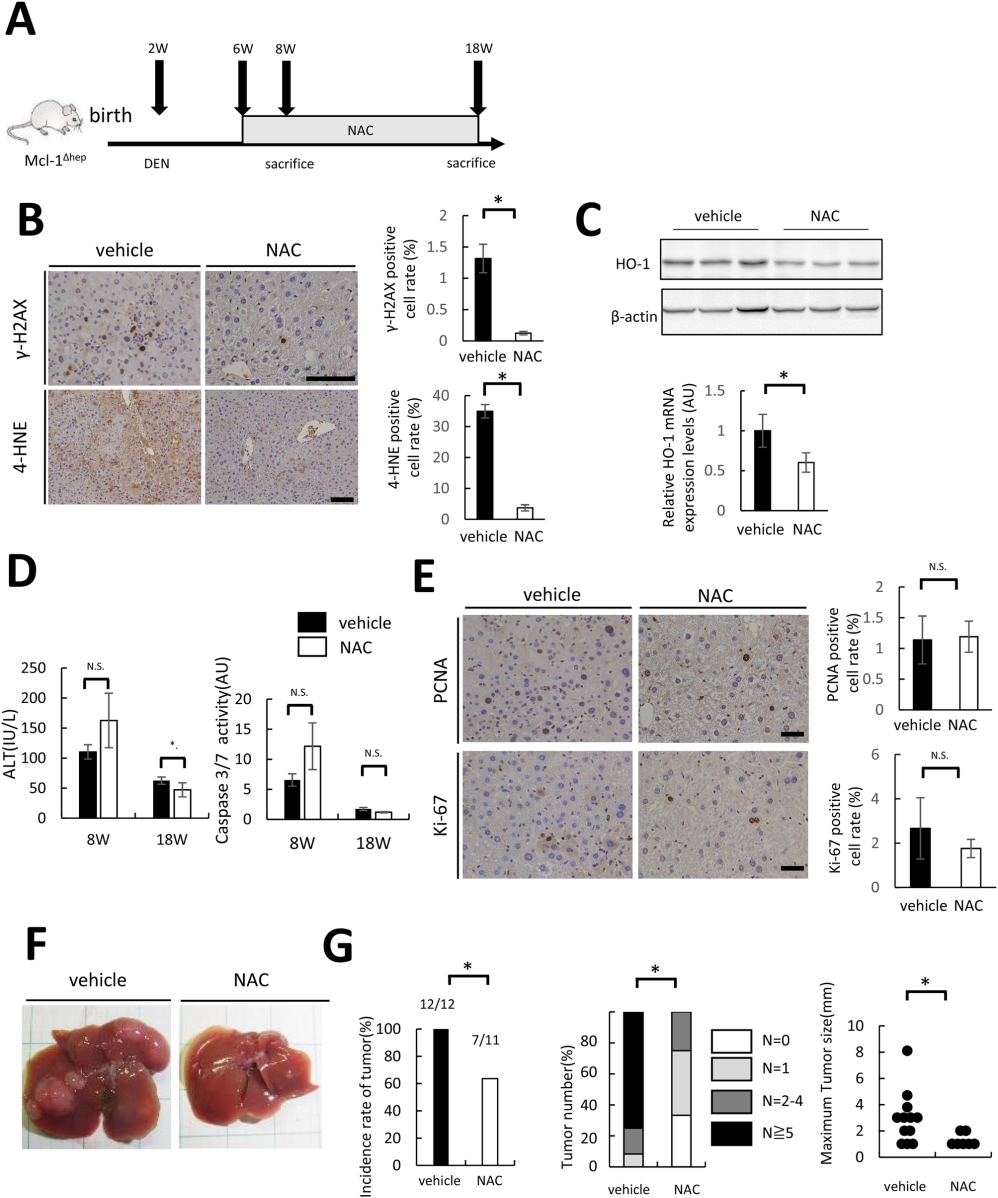
NAC administration significantly decreased liver tumor formation via the improvement of DNA damage in DEN-treated Mcl-1 KO mice. Two-week-old male Mcl-1^Δhep^ mice injected with DEN (20 mg/kg BW) were administered NAC in drinking water from the age of 6 weeks. (**A**) Schematic of the experimental design. (**B**) Representative images of liver sections stained with anti- γ-H2AX or anti-4-HNE antibody (left). The scale bar is 100 µm. Each positive hepatocyte ratio was quantified and is shown as a percentage (right) (N = 7 or more per group, mean ± SEM, *Mann–Whitney* test). (**C**) Serum ALT levels and caspase-3/7 activity at the indicated ages (n = 8 or more per group, mean ± SEM, *Mann–Whitney* test, *p < 0.05). (**D**) Representative immunoblots of HO-1 in the liver (upper), and expression levels of HO-1 by real-time PCR in the liver (lower) (n = 8 per group, mean ± SEM, *Mann–Whitney* test, *p < 0.05). Full-length blots/gels are presented in Supplementary Fig. 3. (**E**) Representative images of liver sections stained with anti-PCNA or Ki-67 antibody (left). The scale bar is 50 µm. Each positive hepatocyte ratio was quantified and is shown as a percentage (right) (n ≥ 7 per group, mean ± SEM, *Mann–Whitney* test). (**F**) Representative images of mice with the indicated genotype at the age of 18 weeks. (**G**) Incidence rate of tumors (*chi-squared* test), incidence rate of tumors by tumor number (*chi-squared* test), maximum size of liver tumors (*Mann–Whitney* test, *p < 0.05).

To examine the significance of oxidative stress on the tumor formation of DEN-injected mice, we administered N-acetyl-L-cysteine (NAC) in drinking water for 12 weeks to DEN-treated Mcl-1 KO mice from the age of 6 weeks, which was 4 weeks after DEN injection. Then, we explored the phenotype of these mice at the age of 18 weeks (Fig. 4A). While there was a significant decrease in the 4-HNE-positive cell ratio and the HO-1 expression levels in the NAC group (Fig. 4B, 4C), there were no significant differences in the serum caspase-3/7 activity between the vehicle group and the NAC group (Fig. 4D). The PCNA- and Ki-67-positive hepatocyte ratios also did not differ between the vehicle group and the NAC group (Fig. 4E). In contrast, NAC treatment significantly decreased the phospho-H2AX-positive hepatocyte ratio (Fig. 4B). While all 12 mice treated with vehicle developed macroscopic liver tumors, only 7 of 11 mice treated with NAC did (Fig. 4F,G). The frequency of multiple liver tumors and the maximum size of liver tumors were significantly decreased in the NAC group compared with the vehicle group (Fig. 4G).

## Discussion

DEN is a well-known chemical carcinogen that induces liver tumors in mice. In the present study, we injected DEN into mice, which was followed by persistent hepatocyte apoptosis by the ablation of Mcl-1, an anti-apoptotic protein. We demonstrated that DEN-induced, B-Raf-driven, liver tumor formation is promoted by persistent hepatocyte apoptosis. The promotion was suppressed by oxidative stress reduction without any change in hepatocyte apoptosis or compensatory liver regeneration.

We previously reported that persistent hepatocyte apoptosis causes gene mutations via oxidative stress, which is related to liver tumor development^18^. In the present study, we demonstrated that persistent hepatocyte apoptosis-induced oxidative stress accelerates tumor formation in livers harboring transformed hepatocytes. Collectively, oxidative stress in livers with persistent hepatocyte apoptosis works at both points of hepatocyte transformation and accelerates tumor formation after acquiring transformation. Gentric et al^22^ reported that antioxidant treatment restored hepatocyte pathological polyploidization, so they concluded that oxidative stress promoted pathological polyploidization, which was suggested to be an early event in the hepatocarcinogenesis of nonalcoholic fatty liver disease (NAFLD). Maeda et al^23^ reported that hepatocyte-specific IkKβ deletion increased oxidative stress and enhanced DEN-induced liver tumor development. In Ma’s report^24^, ROS decreased CD4 + T lymphocytes, which promoted hepatocarcinogenesis in NAFLD livers. Oxidative stress promotes liver tumor formation through various pathways.

It has been reported that DEN-induced liver tumor formation in mice is suppressed by the deficiency of BH3-only protein, Puma^25^, Bid^26^, or Bok^27^. In these reports, based on the results that Puma, Bid or Bok deficiency suppressed both hepatocyte apoptosis and ompensatory liver regeneration after DEN injection, it is speculated that hepatocyte apoptosis or compensatory liver regeneration contributes to the acceleration of DEN-induced liver tumor formation. Consistent with these reports, in the present study, hepatocyte apoptosis efficiently promoted DEN-induced liver tumor formation. However, we clearly demonstrated that the promoting effect was attenuated by antioxidants without any change in hepatocyte apoptosis or liver regeneration. Based on our findings, since hepatocyte apoptosis generates oxidative stress^18^, we speculate that Puma, Bid, and Bok deficiency reduces not only hepatocyte apoptosis but also oxidative stress in the livers, which may be involved in the suppressive mechanism of DEN-induced tumors in Puma-, Bid-, and Bok-deficient mice.

In clinical settings, it has been previously reported that serum ALT levels are a risk factor for HCC incidence in patients with various chronic liver diseases, including viral hepatitis^28^ and nonviral hepatitis^29^. Given that the presence of hepatocyte apoptosis is reflected as high serum ALT, one of the reasons high ALT is a risk for the development of HCC may be that persistent hepatocyte apoptosis accelerates the process from the appearance of transformed cells to tumor formation. From this viewpoint, it is important to aim to normalize ALT in patients with chronic liver disease. Recently, HCV has been eliminated with direct-acting antiviral (DAA) treatment in most cases^30^. However, some genetic or epigenetic changes have been detected in HCV-eliminated cirrhotic livers^31,32^. HBV DNA levels can be maintained at undetectable levels by nucleos(t)ide analog (NA) treatment^33^; however, inserted HBV DNA cannot be eliminated by NA treatment. In these patients already treated or undergoing treatment for chronic viral hepatitis, from a carcinogenic point of view, although ALT elevation due to viral infection is controlled, attention should also be paid to ALT elevation due to other factors, such as metabolic dysfunction-associated fatty liver disease and alcoholic liver disease. For patients whose ALT elevation cannot be controlled, even if the patients’ hepatocytes already have a genetic mutation, treatments with antioxidants may be an option for delaying liver cancer development.

In conclusion, persistent hepatocyte apoptosis accelerates tumor formation in livers harboring DNA-damaged hepatocytes via an increase in oxidative stress, which is independent of compensatory liver regeneration. For these livers, apoptosis no longer plays a positive role in preventing liver tumors but works negatively.

## Materials and methods

### Mice

Hepatocyte-specific Mcl-1 knockout mice (Mcl-1^Δhep^ mice) were generated by crossing Mcl-1^*fl/fl*^ with *albumin-Cre* transgenic mice, as described previously^13^. We injected a single intraperitoneal dose of DEN (Sigma-Aldrich) (20 mg/kg) into 2-week-old male mice to induce a gene alteration in hepatocytes. As a control for DEN injection, we injected PBS. In the NAC treatment group, hepatocyte-specific Mcl-1 KO mice were given NAC (Sigma-Aldrich) water at a dose of 1 g/L from 6 to 8 weeks or 18 weeks and then sacrificed. The mice were maintained in a specific pathogen-free facility and treated with humane care. All mouse experiments were approved by the Animal Care and Use Committee of Osaka University Medical School (30-015), and we performed all mouse experiments in accordance with the approved protocol. All experiments were carried out in compliance with the ARRIVE guideline, the relevant guidelines and regulations.

### HE staining and immunohistochemistry

Freshly dissected liver samples were fixed in 10% formalin for 24 h and embedded in paraffin. For hematoxylin and eosin (HE) staining, 4-µm tissue sections were stained with HE. For immunohistochemistry, cleaved caspase-3, γ-H2AX, 4-HNE, PCNA and Ki-67 were labeled in paraffin-embedded liver sections using an anti-cleaved caspase-3 antibody, anti-PCNA antibody, anti-Ki-67 antibody (Cell Signaling Technology), and anti-4-HNE antibody (Abcam), respectively. The detection of immunolabeled proteins was performed using an avidin–biotin complex with the Vectastain ABC Kit (Vector Laboratories). Four visual fields of a magnified image (× 20 or × 40) were randomly selected for each immune-stained section, and the positive cell ratio was calculated.

### Serum ALT levels and caspase-3/7 activity measurement

For measurement of serum ALT levels and caspase-3/7 activity, blood was collected from the inferior vena cava of the mice. After centrifugation, serum was stored at − 20 °C until use. Serum ALT levels were measured at the Oriental Kobo Life Science Laboratory (Nagahama, Japan). Serum caspase-3/7 activity was measured using a luminescent substrate assay for caspase-3 and caspase-7 (Caspase-Glo assay, Promega, Tokyo, Japan).

### Detection of B-Raf mutation

To detect B-Raf mutations in liver tumors, we performed direct sequencing as previously published. In brief, DNA was extracted from frozen materials with the DNeasy Blood and Tissue Kit (Qiagen). The region that included the B-Raf codon 637 was amplified by PCR using appropriate primers (forward: 5′-gacctcacggtaaaaataggtgac-3′; reverse: 5′-gcaattatgcctggcttacaa-3′) and Platinum PCR SuperMix High Fidelity (Invitrogen). The PCR products were purified with the Genomic DNA Purification Kit (Promega) and sequenced using the BigDye Terminator v3.1 Cycle Sequencing Kit (Thermo Fisher Scientific) on an Applied Biosystems 3730 DNA Analyzer (Thermo Fisher Scientific).

### Western blots

Murine liver lysates were prepared for Western blots as previously described^34^. We used following primary antibodies for detection of the specific proteins: Mcl-1 (#5453, Cell Signaling Technology), Bcl-xL (#2764, Cell Signaling Technology), Bak (#3814, Cell Signaling Technology), Bax (#2772, Cell Signaling Technology), HO-1(#43966, Cell Signaling Technology) and beta actin (A5316, Sigma-Aldrich). We detected the signals using Fusion Solo S (Vilber Lourmat, Collegien, France).

### Real-time PCR

Complementary DNA form murine livers was prepared as previously described^34^.

Quantitative real-time reverse transcription polymerase chain reaction (RT-PCR) were performed using Thunderbird qPCR master mix (Toyobo, Osaka, Japan) and TaqMan probes (Thermo Fisher Scientific). We used following probes; HO-1 (Mm00516005_m1) and Actb (Mm02619580_g1). HO-1 expression levels were normalized to beta-actin expression levels.

### Statistical analysis

Statistical analysis was performed using JMP software. The data are shown as the mean ± standard error of the mean unless otherwise indicated. Comparisons of continuous variables between wild-type mice and Mcl-1^Δhep^ mice were performed using the Mann–Whitney test. Comparisons of noncontinuous variables between wild-type mice and Mcl-1^Δhep^ mice were performed using the *chi-squared* test. *P* < 0.05 was considered significant.

## Supplementary Information

Supplementary Figures.

Supplementary legends.

## Abbreviations

HCC: Hepatocellular carcinoma
HBV: Hepatitis B virus
HCV: Hepatitis C virus
DEN: Diethylnitrosamine
4-HNE: 4-Hydroxy-2-nonenal
HO-1: Heme oxygenase-1
NAC: N-acetyl-L-cysteine
