# Supplementary material for: Persistent hepatocyte apoptosis promotes tumorigenesis from diethylnitrosamine-transformed hepatocytes through increased oxidative stress, independent of compensatory liver regeneration
Source: Sci Rep. 2021 Feb 9;11:3363. doi: 10.1038/s41598-021-83082-7 (PMC7873060; doi:10.1038/s41598-021-83082-7)
Supplement: Supplementary file 1 — Supplementary Figures. [file 41598_2021_83082_MOESM1_ESM.pptx]

## Slide 1
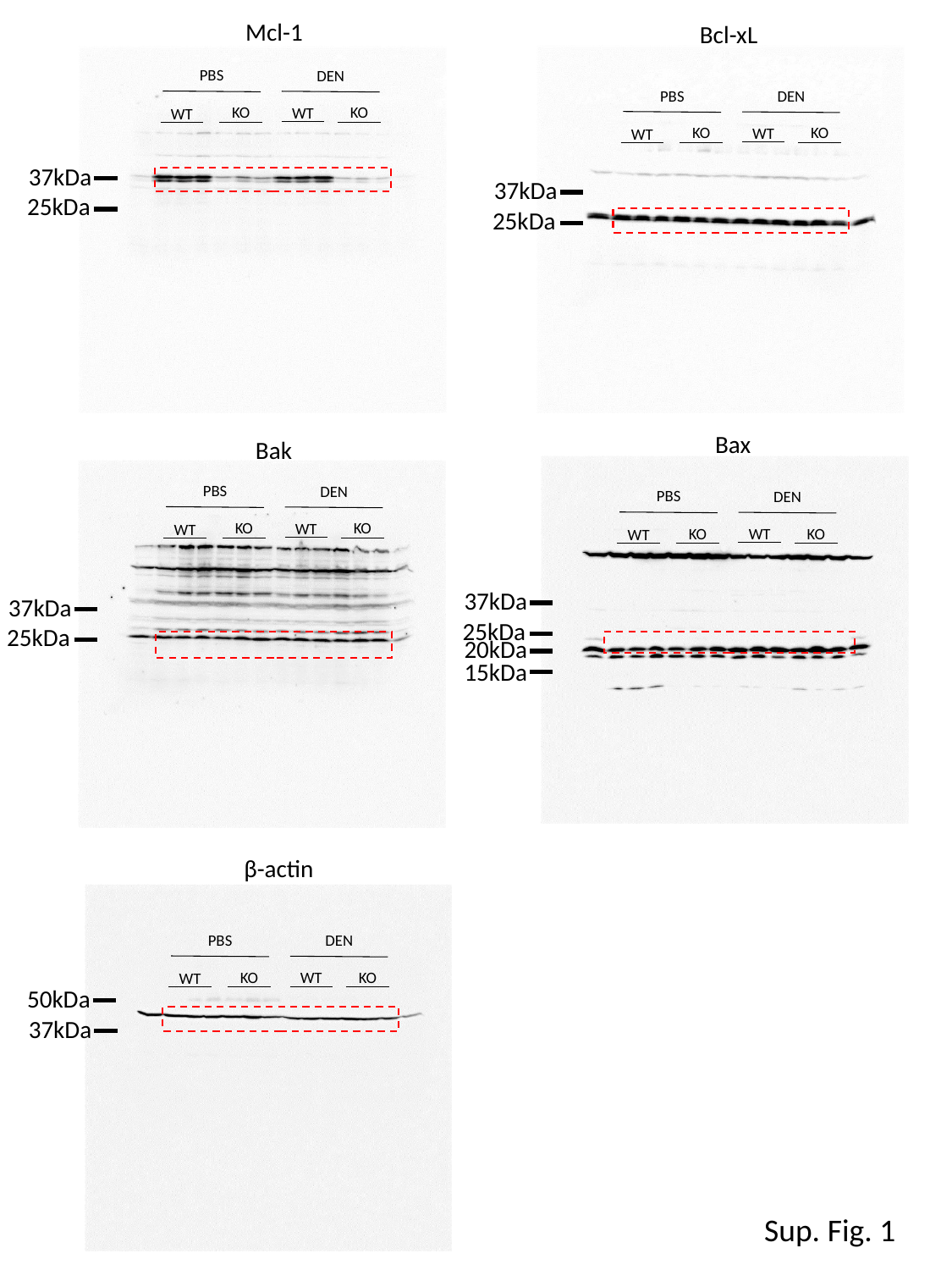

Mcl-1
PBS
DEN
KO
KO
WT
WT
37kDa
25kDa
Bcl-xL
PBS
DEN
KO
KO
WT
WT
37kDa
25kDa
Bax
PBS
DEN
KO
KO
WT
WT
37kDa
25kDa
20kDa
15kDa
Bak
PBS
DEN
KO
KO
WT
WT
37kDa
25kDa
β-actin
PBS
DEN
KO
KO
WT
WT
50kDa
37kDa
Sup. Fig. 1

## Slide 2
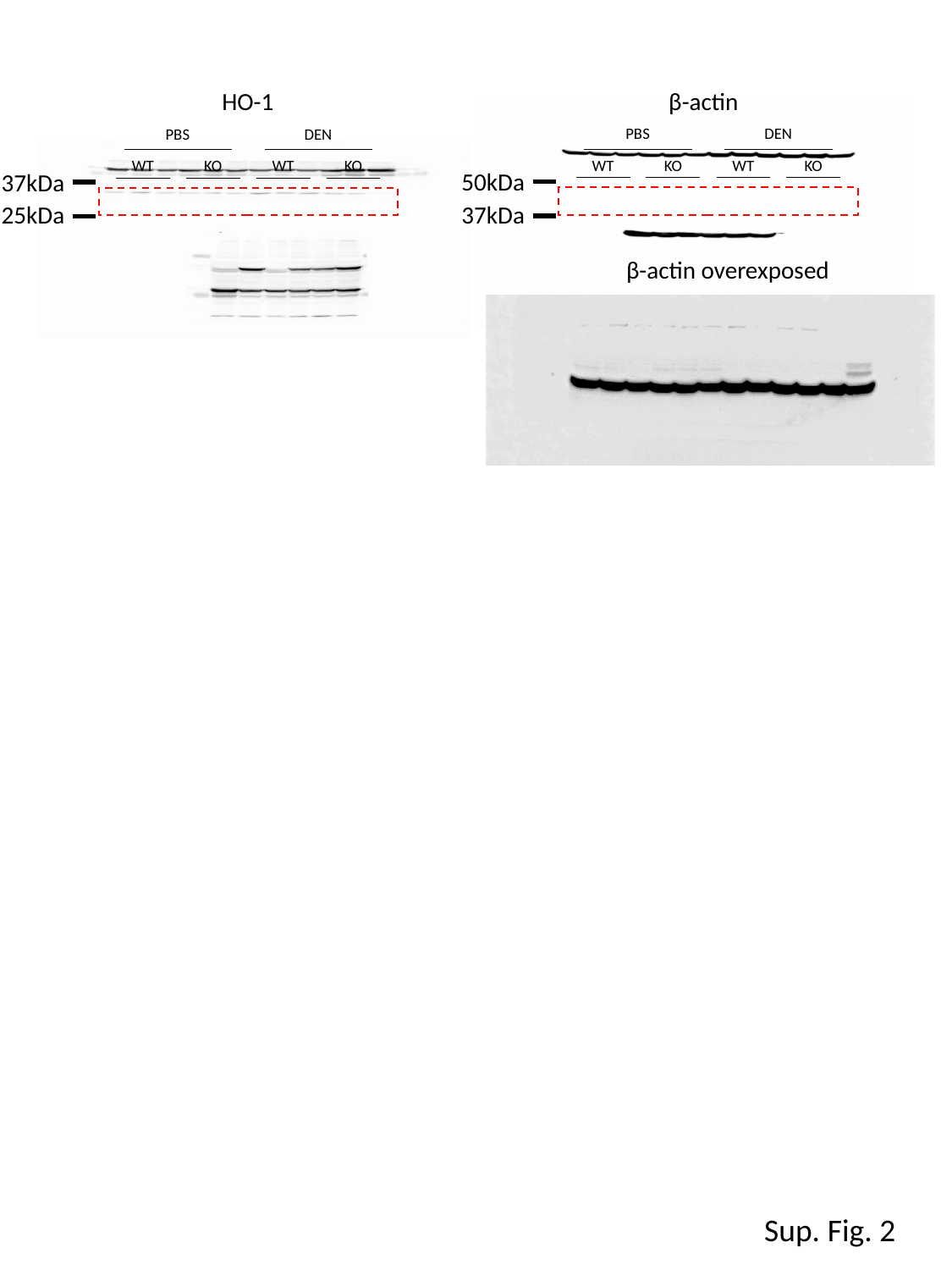

HO-1
β-actin
PBS
DEN
PBS
DEN
WT
KO
WT
KO
WT
KO
WT
KO
50kDa
37kDa
37kDa
25kDa
β-actin overexposed
Sup. Fig. 2

## Slide 3
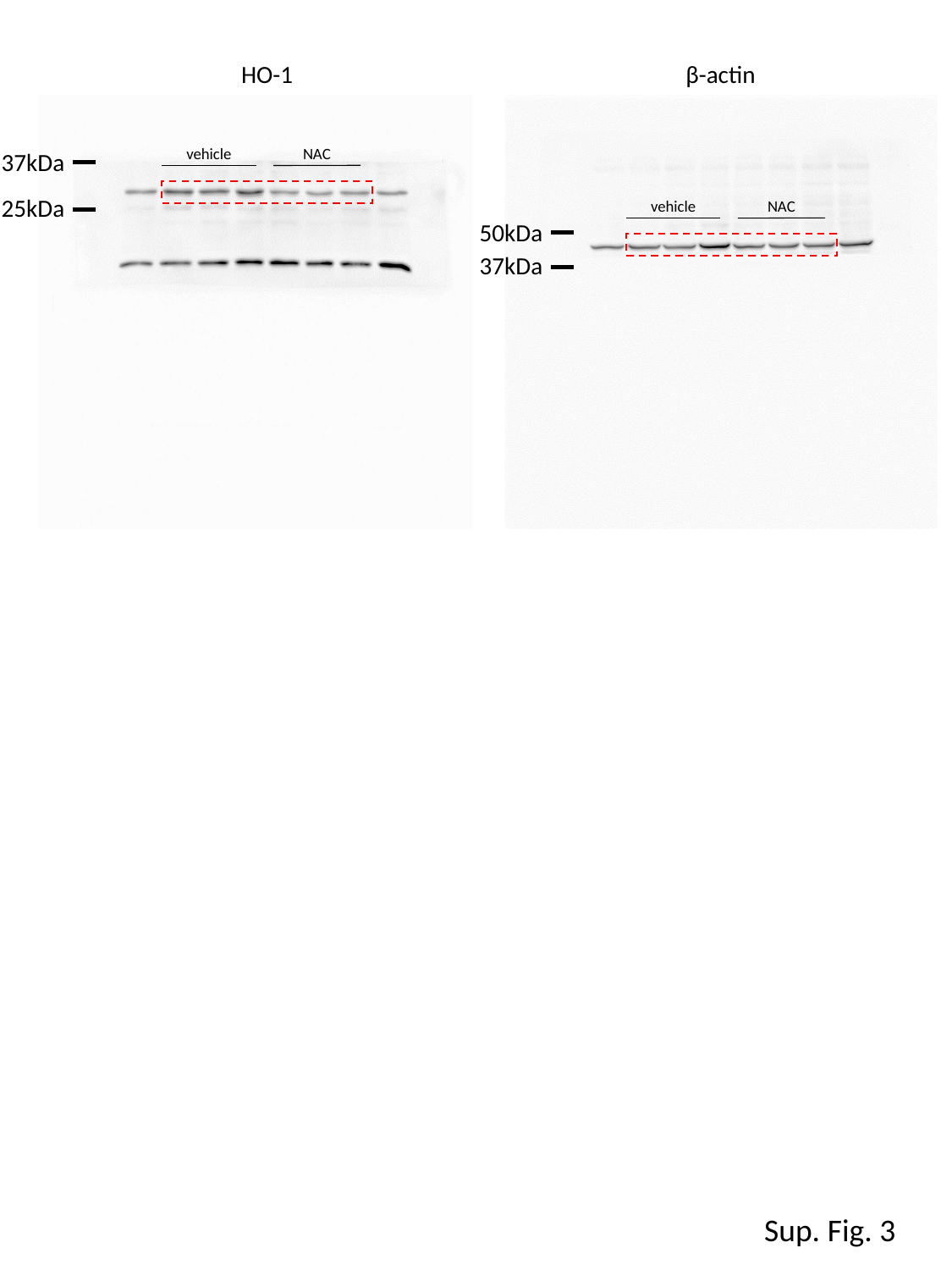

β-actin
HO-1
vehicle
NAC
37kDa
25kDa
vehicle
NAC
50kDa
37kDa
Sup. Fig. 3
