# Supplementary material for: Persistent hepatocyte apoptosis promotes tumorigenesis from diethylnitrosamine-transformed hepatocytes through increased oxidative stress, independent of compensatory liver regeneration
Source: Sci Rep. 2021 Feb 9;11:3363. doi: 10.1038/s41598-021-83082-7 (PMC7873060; doi:10.1038/s41598-021-83082-7)
Supplement: Supplementary file 2 — Supplementary legends. [file 41598_2021_83082_MOESM2_ESM.docx]

**Persistent hepatocyte apoptosis promotes tumorigenesis from diethylnitrosamine-transformed hepatocytes through increased oxidative stress, independent of compensatory liver regeneration**

Yasutoshi Nozaki, Hayato Hikita, Satoshi Tanaka, Kenji Fukumoto, Makiko Urabe, Katsuhiko Sato, Yuta Myojin, Akira Doi, Kazuhiro Murai, Sadatsugu Sakane, Yoshinobu Saito, Takahiro Kodama, Ryotaro Sakamori, Tomohide Tatsumi and Tetsuo Takehara

Department of Gastroenterology and Hepatology

Osaka University Graduate School of Medicine

**Corresponding author**

Tetsuo Takehara

[takehara@gh.med.osaka-u.ac.jp](mailto:takehara@gh.med.osaka-u.ac.jp)

Osaka University Graduate School of Medicine

2-2 Yamadaoka Suita Osaka, Japan

Tel: +81-6-6879-3621; Fax: +81-6-6879-3629

**E-mail addresses**

Yasutoshi Nozaki: y.nozaki@gh.med.osaka-u.ac.jp

Hayato Hikita: hikita@gh.med.osaka-u.ac.jp

Satoshi Tanaka: st0305@gh.med.osaka-u.ac.jp

Kenji Fukumoto: kenjin190301@gh.med.osaka-u.ac.jp

Makiko Urabe: urabe@gh.med.osaka-u.ac.jp

Katsuhiko Sato: k.sato@gh.med.osaka-u.ac.jp

Yuta Myojin: myojin@gh.med.osaka-u.ac.jp

Akira Doi: dokira0820@gh.med.osaka-u.ac.jp

Kazuhiro Murai: k.murai@gh.med.osaka-u.ac.jp

Sadatsugu Sakane: ssakane@gh.med.osaka-u.ac.jp

Yoshinobu Saito: saito0y@gh.med.osaka-u.ac.jp

Takahiro Kodama: t-kodama@gh.med.osaka-u.ac.jp

Ryotaro Sakamori: sakamori@gh.med.osaka-u.ac.jp

Tomohide Tatsumi: tatsumit@gh.med.osaka-u.ac.jp

Tetsuo Takehara: takehara@gh.med.osaka-u.ac.jp

**Supplemental Figure legend**

**Supplemental Figure 1**

Raw images of Western blots in Figure 2A.

**Supplemental Figure 2**

Raw images of Western blots in Figure 3C.

**Supplemental Figure 3**

Raw images of Western blots in Figure 4D.
